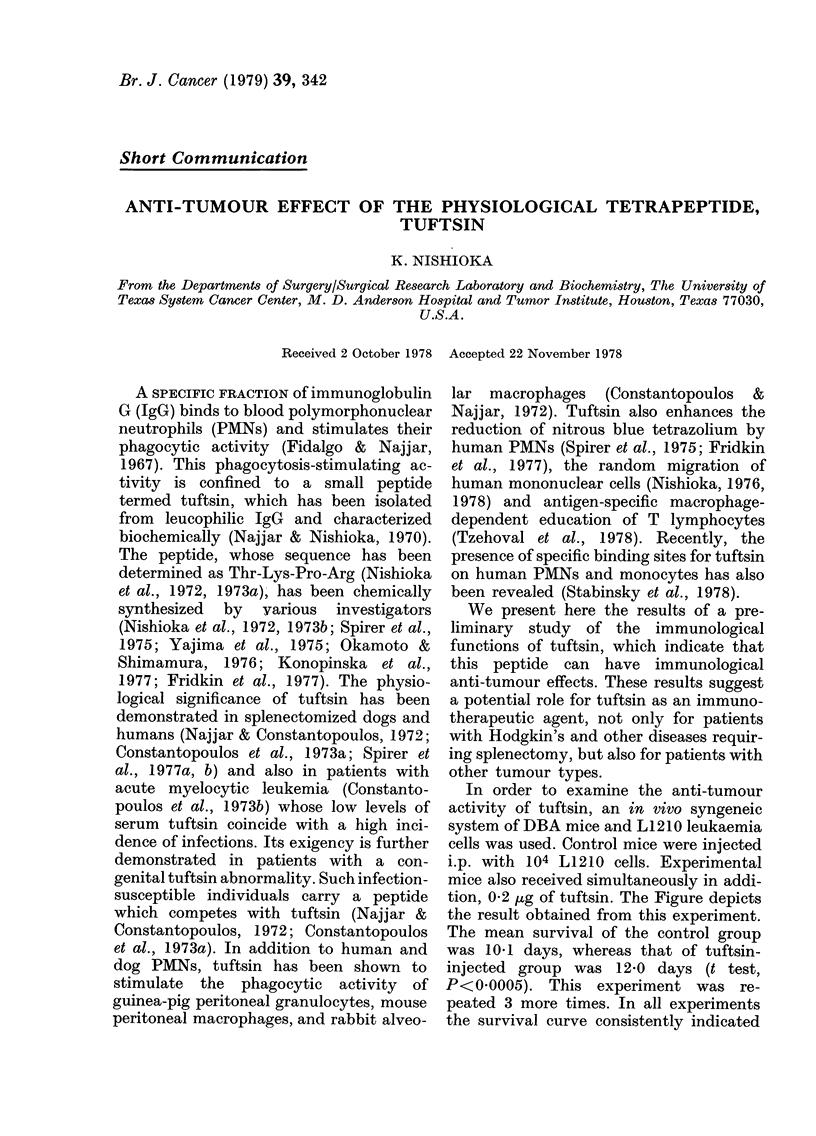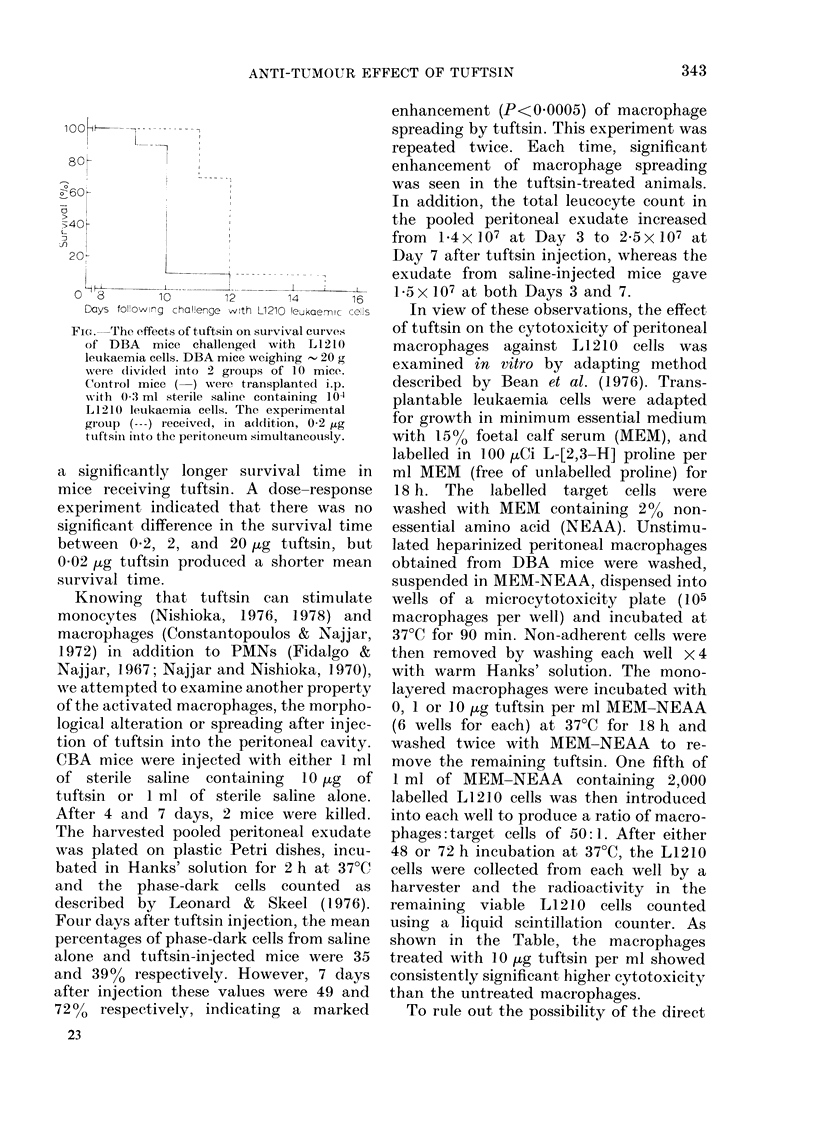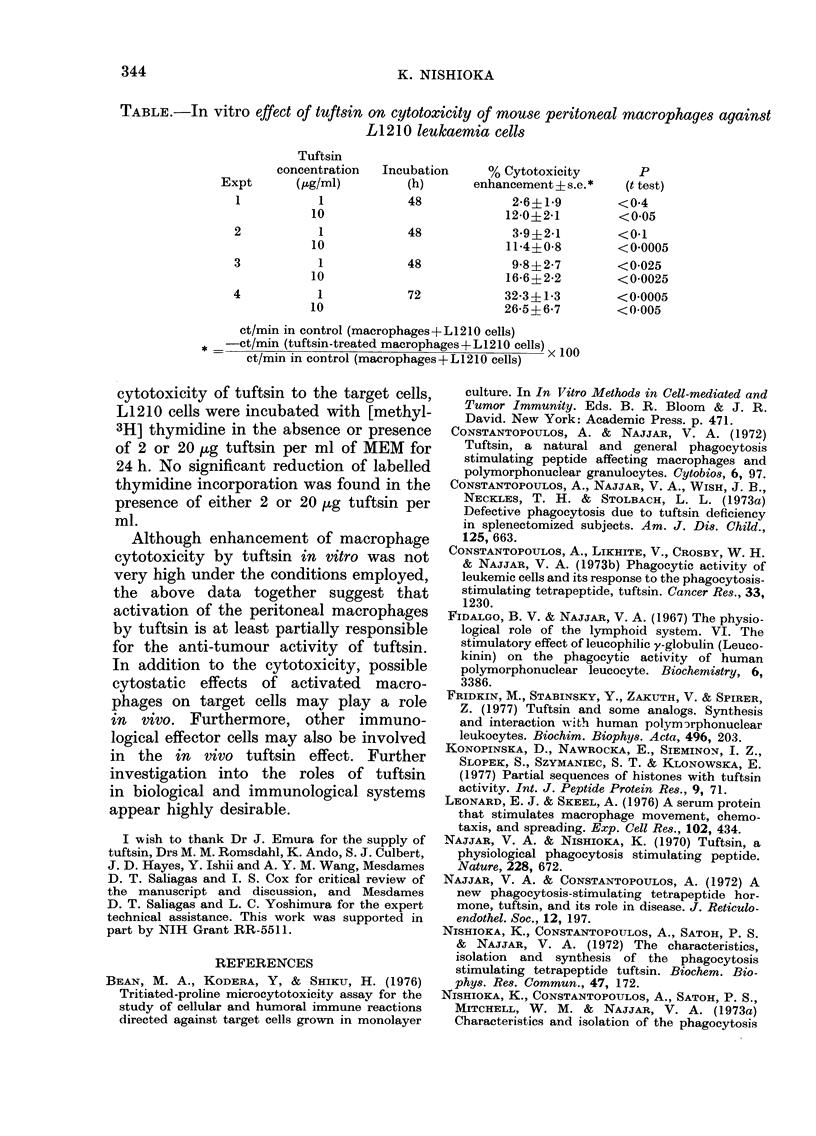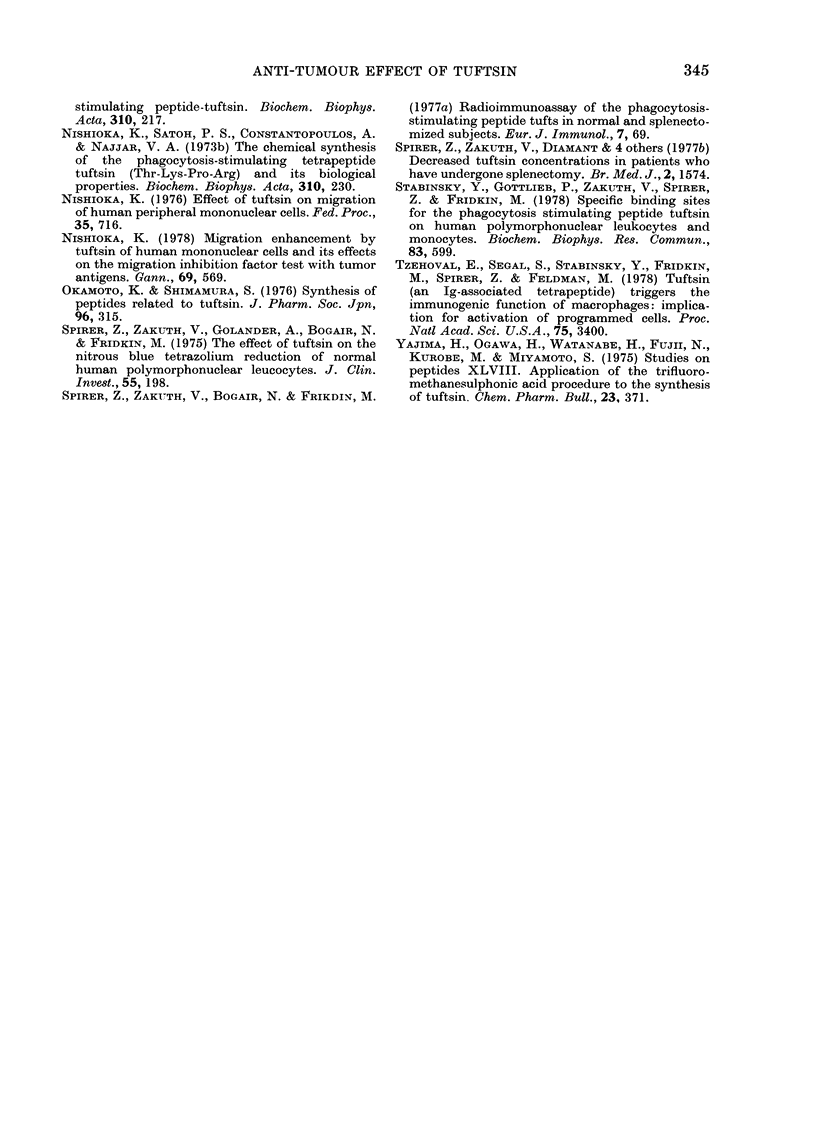# Anti-tumour effect of the physiological tetrapeptide, tuftsin.

**DOI:** 10.1038/bjc.1979.59

**Published:** 1979-03

**Authors:** K. Nishioka


Br. J. Cancer (1979) 39, 342

*Short Communication*

### ANTI-TUMOUR EFFECT OF THE PHYSIOLOGICAL TETRAPEPTIDE, TUFTSIN

K. NISHIOKA

*From the Departments of Surgery/Surgical Research Laboratory and Biochemistry, The University of Texas System Cancer Center, M. D. Anderson Hospital and Tumor Institute, Houston, Texas 77030, U.S.A.*

Received 2 October 1978 Accepted 22 November 1978

A SPECIFIC FRACTION of immunoglobulin G (IgG) binds to blood polymorphonuclear neutrophils (PMNs) and stimulates their phagocytic activity (Fidalgo & Najjar, 1967). This phagocytosis-stimulating activity is confined to a small peptide termed tuftsin, which has been isolated from leucophilic IgG and characterized biochemically (Najjar & Nishioka, 1970). The peptide, whose sequence has been determined as Thr-Lys-Pro-Arg (Nishioka *et al.*, 1972, 1973*a*), has been chemically synthesized by various investigators (Nishioka *et al.*, 1972, 1973*b*; Spirer *et al.*, 1975; Yajima *et al.*, 1975; Okamoto & Shimamura, 1976; Konopinska *et al.*, 1977; Fridkin *et al.*, 1977). The physiological significance of tuftsin has been demonstrated in splenectomized dogs and humans (Najjar & Constantopoulos, 1972; Constantopoulos *et al.*, 1973a; Spirer *et al.*, 1977*a*, *b*) and also in patients with acute myelocytic leukemia (Constantopoulos *et al.*, 1973*b*) whose low levels of serum tuftsin coincide with a high incidence of infections. Its exigency is further demonstrated in patients with a congenital tuftsin abnormality. Such infection-susceptible individuals carry a peptide which competes with tuftsin (Najjar & Constantopoulos, 1972; Constantopoulos *et al.*, 1973*a*). In addition to human and dog PMNs, tuftsin has been shown to stimulate the phagocytic activity of guinea-pig peritoneal granulocytes, mouse peritoneal macrophages, and rabbit alveolar macrophages (Constantopoulos & Najjar, 1972). Tuftsin also enhances the reduction of nitrous blue tetrazolium by human PMNs (Spirer *et al.*, 1975; Fridkin *et al.*, 1977), the random migration of human mononuclear cells (Nishioka, 1976, 1978) and antigen-specific macrophage-dependent education of T lymphocytes (Tzehoval *et al.*, 1978). Recently, the presence of specific binding sites for tuftsin on human PMNs and monocytes has also been revealed (Stabinsky *et al.*, 1978).

We present here the results of a preliminary study of the immunological functions of tuftsin, which indicate that this peptide can have immunological anti-tumour effects. These results suggest a potential role for tuftsin as an immunotherapeutic agent, not only for patients with Hodgkin's and other diseases requiring splenectomy, but also for patients with other tumour types.

In order to examine the anti-tumour activity of tuftsin, an *in vivo* syngeneic system of DBA mice and L1210 leukaemia cells was used. Control mice were injected i.p. with $10^4$ L1210 cells. Experimental mice also received simultaneously in addition, 0·2 $\mu$g of tuftsin. The Figure depicts the result obtained from this experiment. The mean survival of the control group was 10·1 days, whereas that of tuftsin-injected group was 12·0 days (*t* test, $P < 0{\cdot}0005$). This experiment was repeated 3 more times. In all experiments the survival curve consistently indicated

FIG.—The effects of tuftsin on survival curves of DBA mice challenged with L1210 leukaemia cells. DBA mice weighing ~20 g were divided into 2 groups of 10 mice. Control mice (—) were transplanted i.p. with 0·3 ml sterile saline containing $10^4$ L1210 leukaemia cells. The experimental group (---) received, in addition, 0·2 μg tuftsin into the peritoneum simultaneously.

a significantly longer survival time in mice receiving tuftsin. A dose–response experiment indicated that there was no significant difference in the survival time between 0·2, 2, and 20 μg tuftsin, but 0·02 μg tuftsin produced a shorter mean survival time.

Knowing that tuftsin can stimulate monocytes (Nishioka, 1976, 1978) and macrophages (Constantopoulos & Najjar, 1972) in addition to PMNs (Fidalgo & Najjar, 1967; Najjar and Nishioka, 1970), we attempted to examine another property of the activated macrophages, the morphological alteration or spreading after injection of tuftsin into the peritoneal cavity. CBA mice were injected with either 1 ml of sterile saline containing 10 μg of tuftsin or 1 ml of sterile saline alone. After 4 and 7 days, 2 mice were killed. The harvested pooled peritoneal exudate was plated on plastic Petri dishes, incubated in Hanks' solution for 2 h at 37°C and the phase-dark cells counted as described by Leonard & Skeel (1976). Four days after tuftsin injection, the mean percentages of phase-dark cells from saline alone and tuftsin-injected mice were 35 and 39% respectively. However, 7 days after injection these values were 49 and 72% respectively, indicating a marked enhancement ($P<0{\cdot}0005$) of macrophage spreading by tuftsin. This experiment was repeated twice. Each time, significant enhancement of macrophage spreading was seen in the tuftsin-treated animals. In addition, the total leucocyte count in the pooled peritoneal exudate increased from $1{\cdot}4\times10^7$ at Day 3 to $2{\cdot}5\times10^7$ at Day 7 after tuftsin injection, whereas the exudate from saline-injected mice gave $1{\cdot}5\times10^7$ at both Days 3 and 7.

In view of these observations, the effect of tuftsin on the cytotoxicity of peritoneal macrophages against L1210 cells was examined *in vitro* by adapting method described by Bean *et al.* (1976). Transplantable leukaemia cells were adapted for growth in minimum essential medium with 15% foetal calf serum (MEM), and labelled in 100 μCi L-[2,3–H] proline per ml MEM (free of unlabelled proline) for 18 h. The labelled target cells were washed with MEM containing 2% non-essential amino acid (NEAA). Unstimulated heparinized peritoneal macrophages obtained from DBA mice were washed, suspended in MEM-NEAA, dispensed into wells of a microcytotoxicity plate ($10^5$ macrophages per well) and incubated at 37°C for 90 min. Non-adherent cells were then removed by washing each well ×4 with warm Hanks' solution. The monolayered macrophages were incubated with 0, 1 or 10 μg tuftsin per ml MEM–NEAA (6 wells for each) at 37°C for 18 h and washed twice with MEM–NEAA to remove the remaining tuftsin. One fifth of 1 ml of MEM–NEAA containing 2,000 labelled L1210 cells was then introduced into each well to produce a ratio of macrophages:target cells of 50:1. After either 48 or 72 h incubation at 37°C, the L1210 cells were collected from each well by a harvester and the radioactivity in the remaining viable L1210 cells counted using a liquid scintillation counter. As shown in the Table, the macrophages treated with 10 μg tuftsin per ml showed consistently significant higher cytotoxicity than the untreated macrophages.

To rule out the possibility of the direct

TABLE.—In vitro *effect of tuftsin on cytotoxicity of mouse peritoneal macrophages against L*1210 *leukaemia cells*

| Expt | Tuftsin concentration ($\mu$g/ml) | Incubation (h) | % Cytotoxicity enhancement ± s.e.* | *P* (*t* test) |
|---|---|---|---|---|
| 1 | 1 | 48 | 2·6±1·9 | <0·4 |
| | 10 | | 12·0±2·1 | <0·05 |
| 2 | 1 | 48 | 3·9±2·1 | <0·1 |
| | 10 | | 11·4±0·8 | <0·0005 |
| 3 | 1 | 48 | 9·8±2·7 | <0·025 |
| | 10 | | 16·6±2·2 | <0·0025 |
| 4 | 1 | 72 | 32·3±1·3 | <0·0005 |
| | 10 | | 26·5±6·7 | <0·005 |

$$* = \frac{\text{ct/min in control (macrophages + L1210 cells)} - \text{ct/min (tuftsin-treated macrophages + L1210 cells)}}{\text{ct/min in control (macrophages + L1210 cells)}} \times 100$$

cytotoxicity of tuftsin to the target cells, L1210 cells were incubated with [methyl-$^3$H] thymidine in the absence or presence of 2 or 20 $\mu$g tuftsin per ml of MEM for 24 h. No significant reduction of labelled thymidine incorporation was found in the presence of either 2 or 20 $\mu$g tuftsin per ml.

Although enhancement of macrophage cytotoxicity by tuftsin *in vitro* was not very high under the conditions employed, the above data together suggest that activation of the peritoneal macrophages by tuftsin is at least partially responsible for the anti-tumour activity of tuftsin. In addition to the cytotoxicity, possible cytostatic effects of activated macrophages on target cells may play a role *in vivo*. Furthermore, other immunological effector cells may also be involved in the *in vivo* tuftsin effect. Further investigation into the roles of tuftsin in biological and immunological systems appear highly desirable.

I wish to thank Dr J. Emura for the supply of tuftsin, Drs M. M. Romsdahl, K. Ando, S. J. Culbert, J. D. Hayes, Y. Ishii and A. Y. M. Wang, Mesdames D. T. Saliagas and I. S. Cox for critical review of the manuscript and discussion, and Mesdames D. T. Saliagas and L. C. Yoshimura for the expert technical assistance. This work was supported in part by NIH Grant RR-5511.

### REFERENCES

BEAN, M. A., KODERA, Y, & SHIKU, H. (1976) Tritiated-proline microcytotoxicity assay for the study of cellular and humoral immune reactions directed against target cells grown in monolayer culture. In *In Vitro Methods in Cell-mediated and Tumor Immunity*. Eds. B. R. Bloom & J. R. David. New York: Academic Press. p. 471.

CONSTANTOPOULOS, A. & NAJJAR, V. A. (1972) Tuftsin, a natural and general phagocytosis stimulating peptide affecting macrophages and polymorphonuclear granulocytes. *Cytobios*, **6**, 97.

CONSTANTOPOULOS, A., NAJJAR, V. A., WISH, J. B., NECKLES, T. H. & STOLBACH, L. L. (1973*a*) Defective phagocytosis due to tuftsin deficiency in splenectomized subjects. *Am. J. Dis. Child.*, **125**, 663.

CONSTANTOPOULOS, A., LIKHITE, V., CROSBY, W. H. & NAJJAR, V. A. (1973b) Phagocytic activity of leukemic cells and its response to the phagocytosis-stimulating tetrapeptide, tuftsin. *Cancer Res.*, **33**, 1230.

FIDALGO, B. V. & NAJJAR, V. A. (1967) The physiological role of the lymphoid system. VI. The stimulatory effect of leucophilic γ-globulin (Leucokinin) on the phagocytic activity of human polymorphonuclear leucocyte. *Biochemistry*, **6**, 3386.

FRIDKIN, M., STABINSKY, Y., ZAKUTH, V. & SPIRER, Z. (1977) Tuftsin and some analogs. Synthesis and interaction with human polymorphonuclear leukocytes. *Biochim. Biophys. Acta*, **496**, 203.

KONOPINSKA, D., NAWROCKA, E., SIEMINON, I. Z., SLOPEK, S., SZYMANIEC, S. T. & KLONOWSKA, E. (1977) Partial sequences of histones with tuftsin activity. *Int. J. Peptide Protein Res.*, **9**, 71.

LEONARD, E. J. & SKEEL, A. (1976) A serum protein that stimulates macrophage movement, chemotaxis, and spreading. *Exp. Cell Res.*, **102**, 434.

NAJJAR, V. A. & NISHIOKA, K. (1970) Tuftsin, a physiological phagocytosis stimulating peptide. *Nature*, **228**, 672.

NAJJAR, V. A. & CONSTANTOPOULOS, A. (1972) A new phagocytosis-stimulating tetrapeptide hormone, tuftsin, and its role in disease. *J. Reticuloendothel. Soc.*, **12**, 197.

NISHIOKA, K., CONSTANTOPOULOS, A., SATOH, P. S. & NAJJAR, V. A. (1972) The characteristics, isolation and synthesis of the phagocytosis stimulating tetrapeptide tuftsin. *Biochem. Biophys. Res. Commun.*, **47**, 172.

NISHIOKA, K., CONSTANTOPOULOS, A., SATOH, P. S., MITCHELL, W. M. & NAJJAR, V. A. (1973*a*) Characteristics and isolation of the phagocytosis stimulating peptide-tuftsin. *Biochem. Biophys. Acta*, **310**, 217.

NISHIOKA, K., SATOH, P. S., CONSTANTOPOULOS, A. & NAJJAR, V. A. (1973b) The chemical synthesis of the phagocytosis-stimulating tetrapeptide tuftsin (Thr-Lys-Pro-Arg) and its biological properties. *Biochem. Biophys. Acta*, **310**, 230.

NISHIOKA, K. (1976) Effect of tuftsin on migration of human peripheral mononuclear cells. *Fed. Proc.*, **35**, 716.

NISHIOKA, K. (1978) Migration enhancement by tuftsin of human mononuclear cells and its effects on the migration inhibition factor test with tumor antigens. *Gann.*, **69**, 569.

OKAMOTO, K. & SHIMAMURA, S. (1976) Synthesis of peptides related to tuftsin. *J. Pharm. Soc. Jpn*, **96**, 315.

SPIRER, Z., ZAKUTH, V., GOLANDER, A., BOGAIR, N. & FRIDKIN, M. (1975) The effect of tuftsin on the nitrous blue tetrazolium reduction of normal human polymorphonuclear leucocytes. *J. Clin. Invest.*, **55**, 198.

SPIRER, Z., ZAKUTH, V., BOGAIR, N. & FRIKDIN, M. (1977*a*) Radioimmunoassay of the phagocytosis-stimulating peptide tufts in normal and splenectomized subjects. *Eur. J. Immunol.*, **7**, 69.

SPIRER, Z., ZAKUTH, V., DIAMANT & 4 others (1977*b*) Decreased tuftsin concentrations in patients who have undergone splenectomy. *Br. Med. J.*, **2**, 1574.

STABINSKY, Y., GOTTLIEB, P., ZAKUTH, V., SPIRER, Z. & FRIDKIN, M. (1978) Specific binding sites for the phagocytosis stimulating peptide tuftsin on human polymorphonuclear leukocytes and monocytes. *Biochem. Biophys. Res. Commun.*, **83**, 599.

TZEHOVAL, E., SEGAL, S., STABINSKY, Y., FRIDKIN, M., SPIRER, Z. & FELDMAN, M. (1978) Tuftsin (an Ig-associated tetrapeptide) triggers the immunogenic function of macrophages: implication for activation of programmed cells. *Proc. Natl Acad. Sci. U.S.A.*, **75**, 3400.

YAJIMA, H., OGAWA, H., WATANABE, H., FUJII, N., KUROBE, M. & MIYAMOTO, S. (1975) Studies on peptides XLVIII. Application of the trifluoromethanesulphonic acid procedure to the synthesis of tuftsin. *Chem. Pharm. Bull.*, **23**, 371.